# FTIR Detection of Ce^3+^ Sites on Shape-Controlled Ceria Nanoparticles Using Adsorbed ^15^N_2_ as a Probe Molecule

**DOI:** 10.3390/molecules30153100

**Published:** 2025-07-24

**Authors:** Kristina K. Chakarova, Mihail Y. Mihaylov, Bayan S. Karapenchev, Nikola L. Drenchev, Elena Z. Ivanova, Georgi N. Vayssilov, Hristiyan A. Aleksandrov, Konstantin I. Hadjiivanov

**Affiliations:** 1 Institute of General and Inorganic Chemistry, Bulgarian Academy of Sciences, 1113 Sofia, Bulgaria; kristina@svr.igic.bas.bg (K.K.C.); misho@svr.igic.bas.bg (M.Y.M.); bkarapench@uni-sofia.bg (B.S.K.); ndrenchev@svr.igic.bas.bg (N.L.D.); eivanova@svr.igic.bas.bg (E.Z.I.); haa@chem.uni-sofia.bg (H.A.A.); 2 Faculty of Chemistry and Pharmacy, University of Sofia, 1126 Sofia, Bulgaria; gnv@chem.uni-sofia.bg

**Keywords:** adsorption, ceria, nitrogen, FTIR spectroscopy, surface Ce^3+^

## Abstract

Ceria is an important redox catalyst due to the facile Ce^3+^/Ce^4+^ switching at its surface. Therefore, in situ determination of the oxidation state of surface cerium cations is of significant interest. Infrared spectroscopy of probe molecules such as CO holds great potential for this purpose. However, the ability of CO to reduce Ce^4+^ cations is an important drawback as it alters the initial cerium speciation. Dinitrogen (N_2_), due to its chemical inertness, presents an attractive alternative. We recently demonstrated that low-temperature ^15^N_2_ adsorption on stoichiometric ceria leads to the formation of complexes with Ce^4+^ cations on the (110) and (100) planes (bands at 2257 and 2252 cm^−1^, respectively), while the (111) plane is inert. Here, we report results on the low-temperature ^15^N_2_ adsorption on reduced ceria nanoshapes (cubes, polyhedra, and rods). A main band at 2255 cm^−1^, with a weak shoulder at 2254 cm^−1^, was observed. We attributed these bands to ^15^N_2_ adsorbed on Ce^3+^ sites located on edges and corners as well as on {100} facets. In conclusion, ^15^N_2_ adsorbs on the most acidic surface Ce^3+^ sites and enables their distinction from Ce^4+^ cations.

## 1. Introduction

Ceria (CeO_2_) and ceria-based materials are highly versatile, finding applications across various fields including catalysis [1,2], sensors [3], chemical mechanical polishing [4], and advancing biomedicine [5,6]. This versatility stems largely from ceria’s ability to reversibly switch between Ce^4+^ and Ce^3+^ oxidation states, and to accommodate oxygen vacancies without disrupting its stable fluorite lattice structure [7]. As a result, CeO_2_ can readily release or absorb oxygen under varying conditions, making it particularly effective in redox catalysis. A similar mechanism underpins its use in solid oxide fuel cells, where ceria facilitates oxygen ion transport between electrodes [2,7]. Additionally, ceria nanoparticles serve as biological nanozymes, mimicking enzymatic antioxidant activity through redox cycling of Ce^3+^/Ce^4+^ [5,6]. Ceria is also increasingly used in organic synthesis [8], where its catalytic behavior is closely linked to both redox and acid-base surface properties. Therefore, a key challenge in the research of ceria lies in accurately determining the oxidation and coordination state of surface cerium cations under working conditions. However, despite extensive investigation, significant inconsistencies remain in the literature.

Many investigations have been carried out using polycrystalline ceria, which exposes a complex mix of surface facets and structural defects, making it difficult to attribute specific behaviors to individual surface features. To overcome this, recent efforts have focused on nanostructured ceria with well-defined crystallographic planes [9,10,11,12,13,14,15,16,17,18,19,20,21,22,23,24,25], offering model systems that have advanced our understanding of the relationship between surface structure and properties.

The facet-dependent reactivity of ceria is particularly evident in CO oxidation, where the (100) and (110) surfaces are significantly more active than the more stable (111) plane [10,12,13,20,26]. Similar facet-dependent trends are observed in H_2_ oxidation [14,15], soot combustion [27], the water–gas shift (WGS) reaction [16], and reverse WGS reactions [28]. Facet selectivity also governs alcohol conversions; for instance, 2-propanol undergoes dehydrogenation to acetone on {100} facets, whereas dehydration to propene is favored on the (111) surface [22].

Designing efficient CeO_2_-based catalysts requires a deep understanding of surface redox and acid–base properties, which can be probed using advanced characterization techniques applied to well-defined nanostructures. Infrared (IR) spectroscopy is particularly powerful for investigating ceria’s surface chemistry at the molecular level. It allows for the direct observation of surface hydroxyls, adsorbed oxygen species, and common contaminants such as carbonates and nitrates [29,30]. Furthermore, the presence of Ce^3+^ can be tracked via its characteristic spin-orbital f–f transition, typically observed in the 2150–2110 cm^−1^ range [23,31,32,33,34,35,36,37,38,39].

IR spectroscopy of probe molecules is a powerful method for characterizing oxide surfaces. Among these, carbon monoxide (CO) is the most widely used probe [29], but its application to ceria is not without drawbacks. CO can reduce Ce^4+^ to Ce^3+^, forming surface carbonates or hydrogen carbonates [40,41,42], which alter the original surface composition, and carbonate-like features have been detected even after low-temperature (173 K) CO adsorption [21,43]. Moreover, the Ce^3+^-related electronic transition bands overlap with the CO stretching modes, leading to potential misassignments [17,31,44,45,46], unless isotopic labeling is employed [47].

Dinitrogen (N_2_) has emerged as a promising alternative to CO as an infrared probe for studying the surface acidity on oxides [48,49,50,51,52,53,54,55,56,57,58,59]. Its chemical inertness makes it especially attractive for probing ceria surfaces, as it neither oxidizes nor reduces cerium cations, nor does it form surface-bound anions such as carbonates. Thus, N_2_ preserves the original cerium speciation. Furthermore, as a very weak base, N_2_ is unlikely to induce significant surface perturbations.

Although N_2_ is IR-inactive in the gas phase due to its centrosymmetric structure, adsorption lowers its symmetry, rendering the ν(N–N) stretching mode IR-active. The reference frequency for adsorbed N_2_ is proposed at ~2324 cm^−1^, slightly red-shifted from the gas-phase Raman value at 2331 cm^−1^ [49]. When N_2_ interacts with low-valent metal cations such as Cu^+^ [55] or Ni^+^ [58], it forms σ and π bonds that weaken the N≡N bond, leading to a red shift in ν(N–N). In contrast, adsorption onto d^0^ cations like Ce^4+^ occurs via electrostatic interaction [49]. This results in polarization of the molecule and, due to the Stark effect, the N-N stretching frequency is blue shifted. The extent of the shift reflects the polarizing power of the surface cation. Similar is the situation with the Ce^3+^ ions where no back π-donation is expected [25]. Given the weak nature of the interaction in these cases, measurements typically require cryogenic temperatures. It is also worth noting that IR intensity does not directly correlate with the concentration of adsorbed species, as the extinction coefficient depends on polarization. In cases of symmetric adsorption, the IR signal may be nearly undetectable—a phenomenon proposed for Cu–ZSM-5 [55].

Unlike CO, the IR signature of adsorbed N_2_ does not interfere with the gas-phase vibration. However, the ^14^N_2_ band can overlap with CO_2_ gas-phase absorption centered at 2349 cm^−1^. To circumvent this issue, we employed the ^15^N_2_ isotopologue, whose Raman reference frequency is at 2252 cm^−1^.

In this study, we investigate the potential of using ^15^N_2_ as an IR probe for surface characterization of reduced ceria nanoparticles (cubes, polyhedra, and rods). To the best of our knowledge, N_2_ has not yet been applied to this system. To strengthen the assignments, we studied the dosed adsorption of ^15^N_2_, and the co-adsorption of ^15^N_2_ with CO and O_2_. Although the main techniques used were FTIR, TPR, and DFT calculations, the samples were also characterized by XRD, HRTEM, Raman spectroscopy, and XPS. This work completes our previous investigations on ^15^N_2_ adsorption on stoichiometric ceria [59], thus providing a comprehensive view of N_2_ interaction with CeO_2_. It is important to note that detecting both Ce^3+^ and Ce^4+^ sites is essential for ceria-based systems because their importance in catalysis stems mainly from their ability to easily switch between the two cerium oxidation states.

## 2. Results

### 2.1. Basic Characteristics of the Samples

The following three ceria samples with different morphologies were studied: nanocubes (CeO_2_-NC), nanopolyhedra (CeO_2_-NP), and nanorods (CeO_2_-NR). The same materials were used in previous works [24,25,59] and characterized by HRTEM, XRD, TPR, nitrogen adsorption, IR spectroscopy, etc. The key physicochemical characteristics are summarized in Table 1.

#### 2.1.1. Initial Characterization

The XRD patterns of all three samples confirmed their cubic CeO_2_ fluorite structure (space group Fm$\bar{3}$m). HRTEM images showed that the CeO_2_-NC sample consisted of cubic crystals, predominantly terminated by {100} facets. The {110} and {111} facets were also exposed but to a lesser extent. As expected, the CeO_2_-NP sample was characterized by a preferential exposure of the {111} and to a lesser extent the {110} facets at the expense of the {100} facets [24,27,60]. Only a slight reduction in crystallite size and surface area was observed after calcination. In the case of CeO_2_-NR, a high proportion of {110} facets was observed, along with some {111} and {100} facets. The nanorods exhibited the smallest average crystallite size, the highest specific surface area, and the largest void volume between particles. In summary, all three low-index facets were present in each sample, albeit in different proportions. As shown in Table 1, the dominant facets of the CeO_2_-NC, CeO_2_-NP, and CeO_2_-NR were {100}, {111}, and {110}, respectively.

Raman spectroscopy is a widely used technique for characterizing ceria-based systems. This is because Raman spectra are sensitive to some important parameters such as particle size and the presence of defects. The most notable feature in the Raman spectra of stoichiometric ceria is the intense F_2g_ band around 460 ± 5 cm^−1^. Decreasing the crystallite size of CeO_2_ below ca. 50 nm leads to broadening, asymmetry, and red shift of the F_2g_ band [61]. The Raman spectra of our samples are shown in Figure S1 (see Supplementary Materials) and are consistent with these findings. All spectra were dominated by a single band ranging from 462.5 to 457.5 cm^−1^. The lowest frequency was detected in the CeO_2_-NR sample, which had the highest surface area. As expected, the band appeared broad and slightly asymmetric.

Other important features in the Raman spectra of ceria are the D bands. The so-called D2 band, which is observed around 550 cm^−1^, is associated with reduced Ce^3+^ cations [61,62] and also appears with ceria doped with other trivalent cations [62]. This band was not discernible in our spectra, which confirms the lack of Ce^3+^ sites in the calcined samples. Another D band (D1) was observed around 600 cm^−1^ and is associated with stoichiometric defects. This band is usually detected with ceria, having a high specific surface area [61,62,63]. Indeed, the D1/F_2g_ intensity ratio was highest in the CeO_2_-NR sample, which is consistent with its high dispersion.

The samples were also characterized by XPS [24]. The results showed a very low concentration of Ce^3+^ sites (ca. 5 % for all samples), which indicates that most of the cerium cations were in an oxidized state in the calcined samples. Furthermore, we believe that the low amount of Ce^3+^ sites detected by XPS was due to sample reduction during analysis, as recently demonstrated by [64].

#### 2.1.2. Temperature-Programmed Reduction

**Figure 1:**
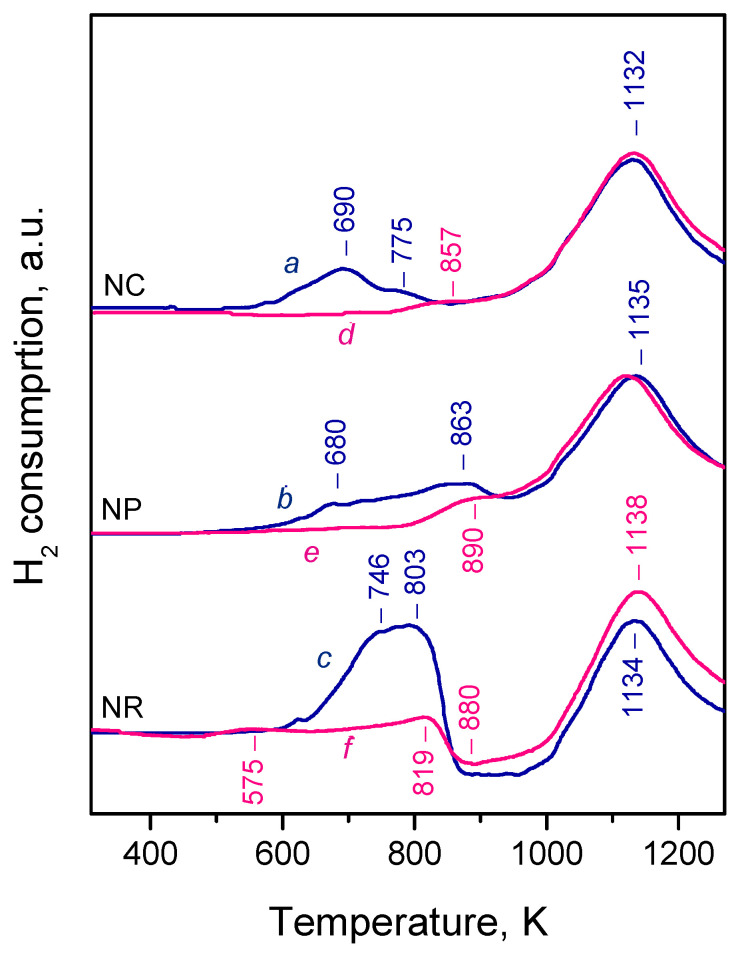
TPR profiles of CeO_2_-NC (*a*,*d*), CeO_2_-NP (*b*,*e*), and CeO_2_-NR samples (*c*,*f*). Patterns *a–c*: pre-oxidized samples; patterns *d–f*: samples pre-reduced at 773 K (see text for details).

Consistent with previous studies [14,21,23,27,38,44,45,46,61,62,63], the H_2_-TPR profiles of our ceria samples were bimodal, showing distinct low-temperature (680–863 K) and high-temperature (1132–1135 K) peaks (Figure 1, patterns *a–c*). The low-temperature peaks depend on the sample morphology and are typically associated with a reduction of surface Ce^4+^ cations, though some reports suggest a contribution from subsurface layers as well [27,65]. The high-temperature peak, nearly identical across all samples, is attributed to a reduction of the bulk.

Among the three samples, CeO_2_-NC exhibited the most easily reducible surface, with a dominant low-temperature peak at 690 K, evidently corresponding to the highly exposed {100} facets. A similar, though less intense, peak was observed with CeO_2_-NP, consistent with its lower {100} facet exposure. Additionally, a peak at 863 K was detected with this sample and could be related to the {111} facets, as recently reported [64]. This aligns with findings that oxygen vacancy formation energy is highest for the (111) plane [66].

The TPR profile of CeO_2_-NR is more complex. In addition to reduction peaks at 746 and 803 K, a broad negative feature appeared between 850 and 1020 K and is attributed to the desorption of dissolved hydrogen [23,61,62]. Therefore, reciprocal component(s) due to hydrogen dissolution should contribute to the positive peaks at a lower temperature. The high hydrogen uptake observed for CeO_2_-NR likely resulted from its high surface area and/or significant exposure of {110} facets. While hydrogen dissolution/desorption cannot be excluded in the other samples, the signals were not clearly discernible.

The amounts of hydrogen consumed during the reduction of the samples up to 773 K and those corresponding to the low-temperature peaks are presented in Table S1 (see Supplementary Materials).

To probe the redox behaviour further, TPR was performed on samples that had been pre-reduced up to 773 K. The aim was to assess whether any surface or subsurface Ce^4+^ sites remained. For CeO_2_-NC (Figure 1, pattern *d*), the initial low-temperature peak nearly disappeared, with only a minor feature around 857 K remaining, possibly due to Ce^4+^ on {111} facets. A similar observation was made for CeO_2_-NP (Figure 1, pattern *e*). Only a weak feature around 890 K was registered in the low-temperature region and can be associated with residual Ce^4+^ cations on the {111} facets.

CeO_2_-NR again showed a distinct behavior. A new peak appeared at 575 K, absent in the oxidized sample, and was attributed to hydrogen dissolution. This process is known to require the presence of Ce^3+^ sites and oxygen vacancies [67], which justify the appearance of these peaks with the reduced sample at a relatively low temperature. However, this peak was relatively weak and did not account for the more intense negative peak around 880 K. Therefore, a second positive peak at 819 K was also ascribed to hydrogen dissolution. Accordingly, in the oxidized sample, the first peak at 746 K can be assigned to Ce^4+^ reduction, and the second (803 K) to hydrogen dissolution.

Despite differing experimental conditions (higher H_2_ pressure, longer reduction time, and static conditions in IR experiments), we show in the discussion that the TPR results suggest that, under the IR reduction protocol, nearly all surface Ce^4+^ sites on CeO_2_-NC and CeO_2_-NR were reduced. A small fraction of non-reduced Ce^4+^ sites remained on CeO_2_-NP.

#### 2.1.3. Background IR Spectra

**Figure 2:**
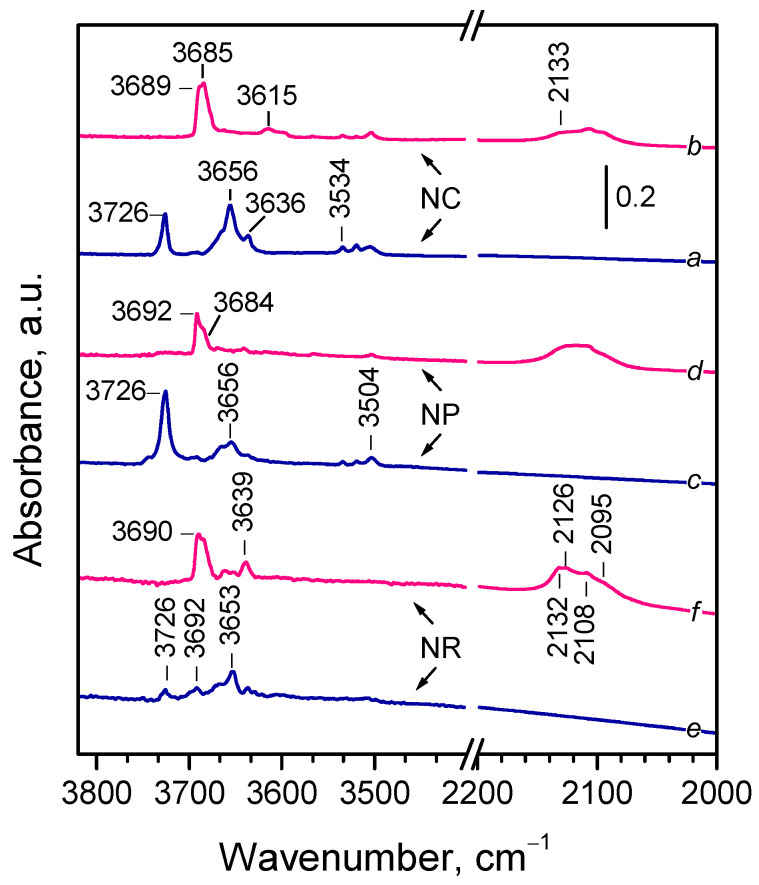
Background FTIR spectra of CeO_2_-NC, CeO_2_-NP, and CeO_2_-NR samples. Spectra are recorded at 100 K in the presence of 2 mbar He with oxidized (*a*,*c*,*e*) and reduced (*b*,*d*,*f*) samples.

In situ FTIR spectra of oxidized and reduced samples recorded at 100 K are compared in Figure 2, focusing on the OH stretching and the Ce^3+^ electronic transition regions. Note that, compared with room temperature, hydroxyl bands appeared at slightly higher wavenumbers (by 3–4 cm^−1^) [24] and the different components of the Ce^3+^ electronic band were well resolved [23].

The infrared spectra of all oxidized samples (Figure 2, spectra *a*,*c*,*e*) contained three groups of OH bands. A sharp band at 3726 cm^−1^ was assigned to terminal Ce^4+^–OH groups [23,24,32,33,35,38,68,69,70,71]. This band was weak with the CeO_2_-NR sample. A series of bands between 3668 and 3636 cm^−1^, and a weak band at 3692 cm^−1^ (only for CeO_2_-NR), were attributed to bridging hydroxyls [23,24,32,33,35,38,44,67,68,69,70,71]. Additionally, low-intensity bands in the 3534–3504 cm^−1^ range were assigned to an oxyhydroxide phase [23,24,32,33,38,68,70,71]. Although spectral details varied, all samples showed features typical of oxidized ceria. Notably, these OH groups were resistant to evacuation at 773 K, suggesting they are associated with defect sites (e.g., edges, corners) rather than flat crystal planes.

Bands due to residual carbonates [32,35] were detected in the 1574–1290 cm^−1^ region with the CeO_2_-NC and CeO_2_-NR samples, but were nearly absent with CeO_2_-NP, in agreement with previous findings [71]. No Ce^3+^ electronic transition bands were detected with the oxidized samples, indicating the absence of reduced Ce^3+^ sites, also supported by their pale yellow color.

The hydroxyl spectra of the reduced samples (Figure 2, spectra *b*,*d*,*f*) were simpler, dominated by bands between 3692 and 3684 cm^−1^, typical of bridging hydroxyls on reduced ceria [23,32,33,35,38,44,66,68,69,72,73]. CeO_2_-NR additionally displayed weak bands at 3662 and 3639 cm^−1^. No terminal OH groups were detected and the oxyhydroxide-related bands were significantly weakened.

All reduced samples exhibited composite Ce^3+^ electronic bands with at least four well resolved components at 2133, 2126, 2108–2106, and 2094 cm^−1^ with varying intensities. A magnified view is shown in Figure S2. Notably, the 2133 cm^−1^ component was most intense with the CeO_2_-NR sample.

### 2.2. FTIR Study of ^15^N_2_ Adsorption

#### 2.2.1. General Observations

**Figure 3:**
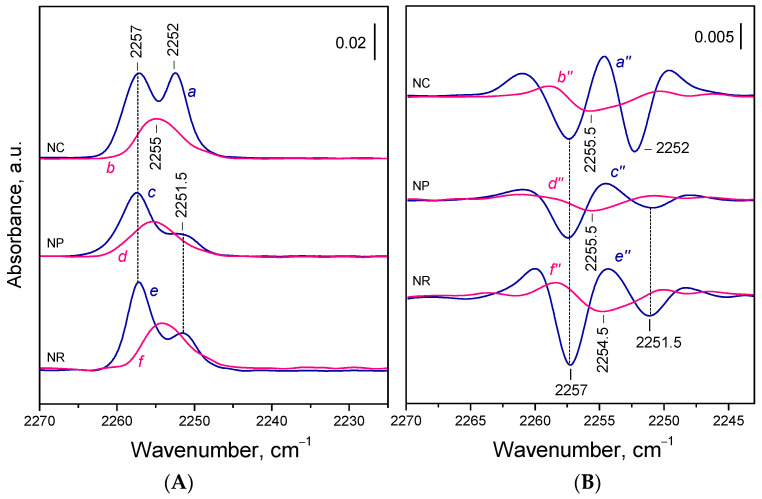
(**A**) FTIR spectra of ^15^N_2_ (0.5 mbar) adsorbed at 100 K on CeO_2_-NC (*a*,*b*), CeO_2_-NP (*c*,*d*), and CeO_2_-NR (*e*,*f*). Oxidized samples (*a*,*c*,*e*) and reduced samples (*b*,*d*,*f*). (**B**) Second derivatives of the spectra presented in panel A.

As previously reported [59], adsorption of ^15^N_2_ on the oxidized ceria gave rise to two distinct IR bands centered at 2257 and 2252–2251.5 cm^−1^ (Figure 3A, spectra *a*,*c*,*e*). The relative intensities of these bands varied among the samples and reflected differences in the exposure of specific crystal facets. The band at 2252 cm^−1^ was assigned to ^15^N_2_ adsorbed on Ce^4+^ sites on the {100} facets, while the 2257 cm^−1^ band was related to Ce^4+^ sites on the {110} facets. The contribution of species formed on edge sites to the latter band was also considered. The Ce^4+^ sites from the {111} facets were too weakly electrophilic to bind ^15^N_2_.

When ^15^N_2_ was adsorbed on the reduced samples, one principal band at ca. 2255 cm^−1^ appeared in all three cases (Figure 3A, spectra *b*,*d*,*f*). This band was absent from oxidized samples and was therefore assigned to ^15^N_2_ species formed on Ce^3+^ sites. Its intensity was 40–60% of the total intensity observed in oxidized samples. No bands typical of ^15^N_2_ adsorbed on stoichiometric samples were detected, which confirms the full surface reduction of the Ce^4+^ cations on the {100} and {110} facets.

#### 2.2.2. Adsorption of ^15^N_2_ on Reduced Ceria Nanocubes (CeO_2_-NC)

**Figure 4:**
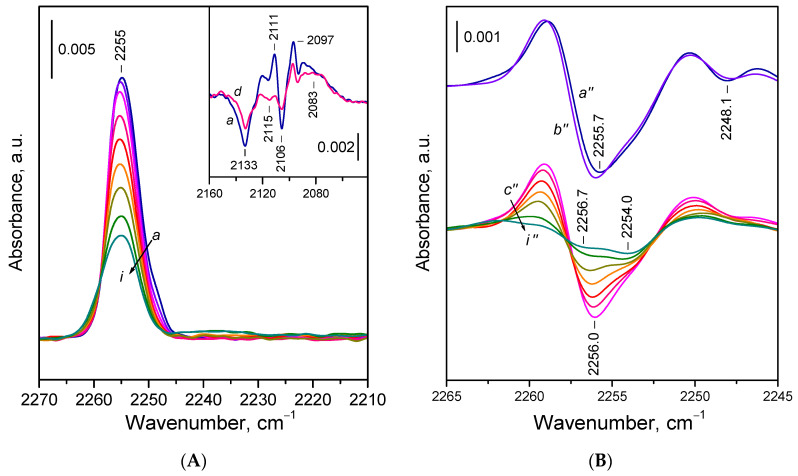
(**A**) FTIR spectra of ^15^N_2_ adsorbed at 100 K on reduced CeO_2_-NC sample. Equilibrium ^15^N_2_ pressure of 0.5 mbar (*a*) and evolution of the spectra under dynamic vacuum (*b–i*). The inset shows the changes in the Ce^3+^ electronic band caused by ^15^N_2_ adsorption. (**B**) Second derivatives of the spectra presented in Panel **A**.

Let us now examine the adsorption of ^15^N_2_ on the reduced CeO_2_-NC sample in more detail. Exposure of the sample to 0.5 mbar of ^15^N_2_ at 100 K resulted in the appearance of a band centered around 2255 cm^−1^, accompanied by a weak shoulder near 2248 cm^−1^ (Figure 4A, spectrum *a*). Upon evacuation, the shoulder disappeared rapidly, and the main band decreased in intensity. The shoulder at 2248 cm^−1^ is attributed to ^15^N_2_ weakly polarized by surface OH groups. In the hydroxyl stretching region, only minor changes were observed and difference spectra revealed a red shift of an OH band at ca. 3685 cm^−1^ by approximately 13 cm^−1^, indicating a very weak interaction, consistent with the negligible acidity of the OH groups of Ce^3+^ cations. Consequently, the 2248 cm^−1^ band was easily removed by evacuation. As will be discussed later, ^15^N_2_ adsorbed on basic sites might also contribute to this band.

Second derivative analysis (Figure 4B, spectra *c*׳׳–*i*׳׳) shows that the band remaining after destruction of the OH ^15^N_2_ species is composite, comprising two components centered at ca. 2256 and 2254 cm^−1^. The higher frequency component shifted slightly towards 2257 cm^−1^ as coverage decreased, while the maximum of the lower frequency component remained unchanged. The component at 2254 cm^−1^ was more resistant to evacuation, indicating more stable species.

Interestingly, the stability contradicts the expected trend in which stronger Ce^3+^–^15^N_2_ interactions (i.e., more stable adducts) would result in a higher frequency band. A possible explanation is that the 2254 cm^−1^ band corresponds to species in which the N_2_ molecule is bound by both ends, but predominantly through one nitrogen atom. This binding mode leads to lower polarization of the N_2_ molecule but enhances overall stability. However, based on the DFT studies (see below), we propose an alternative assignment of the band, namely to ^15^N_2_ bridging two Ce^3+^ sites by one ^15^N atom.

Close inspection of the spectra recorded during the final stages of evacuation (Figure 4A, spectra *h*,*i*) reveals the emergence of a faint shoulder around 2259 cm^−1^ and a broad absorption between 2245 and 2230 cm^−1^. These features are associated with minor oxygen uptake and partial reoxidation of few Ce^3+^ sites. The component at 2259 cm^−1^ is attributed to ^15^N_2_ on Ce^4+^ sites, while the bands between 2245 and 2230 cm^−1^ are attributed to the first overtone of superoxide O_2_^−^ species. These assignments were confirmed by the addition of small amounts of O_2_ to the system (details not reported here).

Adsorption of ^15^N_2_ also induced changes in the Ce^3+ 2^F_5/2_ → ^2^F_7/2_ electronic transition band (Figure 4A). Difference spectra showed two well-defined minima at 2133 and 2106 cm^−1^, alongside a broad positive band peaking around 2083 cm^−1^. Two sharp maxima at 2111 and 2097 cm^−1^ were also present, though they may have resulted from an overlap between a broad positive band at ~2104 cm^−1^ and a sharp negative band at 2106 cm^−1^. This interpretation is supported by spectra registered with the CeO_2_-NR sample (see below). Upon evacuation, the spectra gradually reverted to the original state.

#### 2.2.3. Adsorption of ^15^N_2_ on Reduced Ceria Nanopolyhedra (CeO_2_-NP)

**Figure 5:**
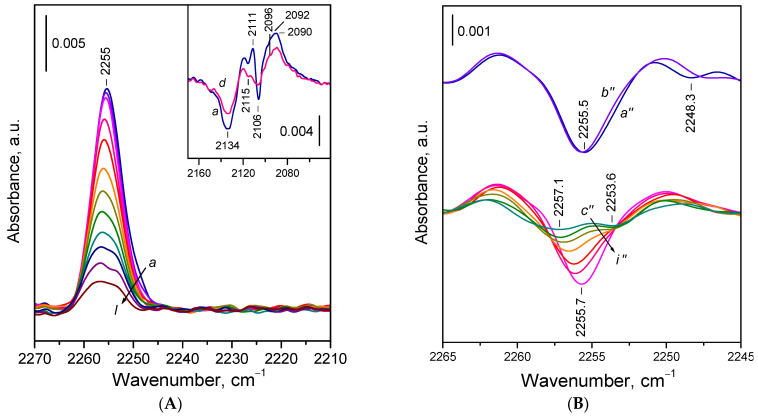
(**A**) FTIR spectra of ^15^N_2_ adsorbed at 100 K on reduced CeO_2_-NP. Equilibrium ^15^N_2_ pressure of 0.5 mbar (*a*) and evolution of the spectra under dynamic vacuum (*b–l*). The inset shows the changes in the Ce^3+^ electronic band caused by ^15^N_2_ adsorption. (**B**) Second derivatives of the spectra presented in Panel **A**.

The FTIR spectra of ^15^N_2_ adsorbed on the reduced CeO_2_-NP sample are presented in Figure 5. Similar to CeO_2_-NC, a single dominant band appeared near 2255 cm^−1^, composed of two components. Compared with CeO_2_-NC, the lower frequency component was less pronounced and appeared at slightly lower wavenumbers. The changes in the Ce^3+^ electronic transition band upon ^15^N_2_ adsorption are comparable to those observed for CeO_2_-NC (Figure 5A).

#### 2.2.4. Adsorption of ^15^N_2_ and ^14^N_2_ on Reduced Ceria Nanorods (CeO_2_-NR)

**Figure 6:**
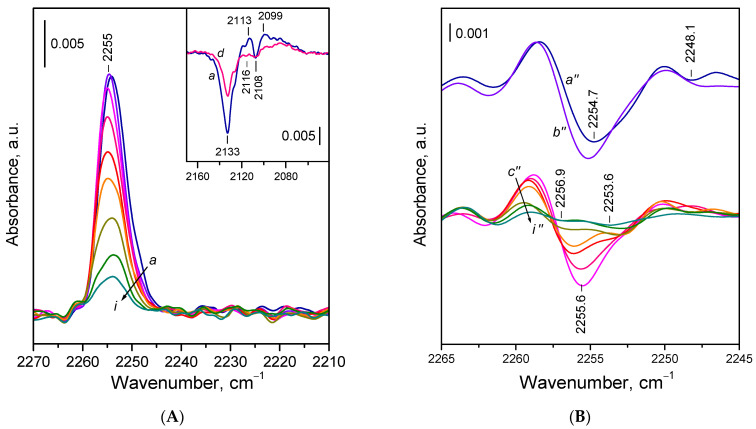
(**A**) FTIR spectra of ^15^N_2_ adsorbed at 100 K on reduced CeO_2_-NR. Equilibrium ^15^N_2_ pressure of 0.5 mbar (*a*) and evolution of the spectra under dynamic vacuum (*b–i*). The inset shows the changes in the Ce^3+^ electronic band caused by ^15^N_2_ adsorption. (**B**) Second derivatives of the spectra presented in Panel **A**.

The general spectral pattern of ^15^N_2_ adsorption on reduced CeO_2_-NR (Figure 6) was similar to that observed for the other samples. A notable difference was the much more pronounced negative band at 2134–2133 cm^−1^. In addition, the component at ca 2254 cm^−1^ was less intense compared with the CeO_2_-NC sample.

**Figure 7:**
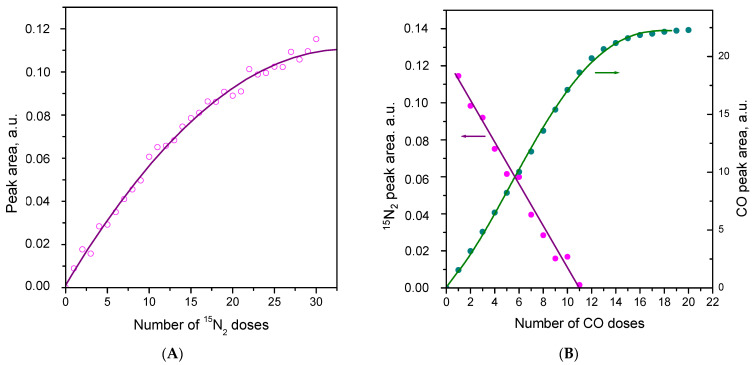
(**A**) Dependence of the integral intensity of the ^15^N_2_ band at 2255 cm^−1^ on the amount of ^15^N_2_ added to CeO_2_-NP at 100 K. (**B**) Dependencies of the integral intensities of the ^15^N_2_ and CO bands on the amount of CO added at 100 K to the system 0.5 mbar ^15^N_2_/CeO_2_-NR.

To check for the eventual existence of IR-invisible adsorbed dinitrogen, we studied successive adsorption of small doses of ^15^N_2_. The respective IR spectra are shown in Figure S3, while the dependence of the integral intensity of the ^15^N_2_ stretching band on the added amount of dinitrogen is depicted on Figure 7A. Inspection of Figure 7A shows that the 2255 cm^−1^ band began to develop after the first doses of added ^15^N_2_, and the dependence at low coverage was nearly linear. This clearly indicates the absence of a stronger IR-invisible adsorption form because, in such a case, the 2255 cm^−1^ band should have begun to develop with some delay. However, the results do not exclude the existence of a weakly bound invisible ^15^N_2_.

It is also to be noted that no negative band at 2133 cm^−1^ appeared after adsorption of the first several doses of ^15^N_2_ (Figure S3). This band started to develop at high coverages and was of significant intensity only under some ^15^N_2_ equilibrium pressure. Simultaneously a negative OH band developed at 3685 cm^−1^. These observations imply that the 2133 cm^−1^ band or at least part of it is associated with hydroxylated Ce^3+^ sites.

To obtain more information on this issue, we studied the gradual displacement of adsorbed N_2_ by CO. The FTIR spectra are shown in Figure S4, and the dependencies of the ^15^N_2_ and carbonyl band intensities on the amount of CO added into the system are presented in Figure 7B. As can be seen, the intensity of the ^15^N_2_ band decreased linearly with the amount of added CO and it disappeared completely before the carbonyl band fully developed. Thus, the results practically exclude the possibility of the existence of an invisible ^15^N_2_ with an adsorption strength comparable to that of the 2255 cm^−1^ adsorption form. For more details, see the discussion section.

It should also be noted that, after replacing all adsorbed ^15^N_2_, a carbonyl band around 2147 cm^−1^ developed. This band has been attributed to CO complexes on the reduced {110} facets [25]. Thus, the result indicates that N_2_ is not adsorbed on these facets.

We also considered the possibility of the existence of some ^15^N_2_ bands that lie in the region of the Ce^3+^ electronic transition and are masked by it. These bands would presumably be due to molecules that are almost parallel to the surface. If such bands exist, they should appear around 2160 cm^−1^ when ^14^N_2_ is adsorbed. However, the experiment with ^14^N_2_ revealed the appearance of only one band around 2133 cm^−1^, which clearly indicates the absence of any ^15^N_2_ bands in the 2140–2090 cm^−1^ region.

Finally, we studied the low-temperature co-adsorption of ^15^N_2_ and O_2_. As already described, the adsorption of ^15^N_2_ results in the appearance of a bandwith two components at 2256 and 2254 cm^−1^ (See Figure S5, spectrum a, and the corresponding second derivative *a”*). Dosing oxygen to the system led to the erosion of these two bands and the appearance of (i) bands at 2258.5 and 2251.7 cm^−1^, typical of ^15^N_2_ adsorbed on Ce^4+^ sites, and (ii) weak bands at 2241.5 and 2232.5 cm^−1^, due to the first overtone of the O–O stretching modes of adsorbed superoxide (O_2_^−^) species [23]. It is important to note that at the end of the process the bands characteristic of ^15^N_2_ adsorbed on reduced Ce^3+^ sites totally disappeared, which indicates the full oxidation of the Ce^3+^ adsorption sites to Ce^4+^ (Figure S5, spectrum *b* and the corresponding second derivative *b’’*).

In conclusion, two types of dinitrogen complexes of Ce^3+^ cations consistently appeared across all reduced samples, though with varying intensity ratios. Some Ce^3+^ sites were too weakly acidic to adsorb ^15^N_2_, even at 100 K.

### 2.3. DFT Modeling of N_2_ Adsorption on Ceria

To further understand the adsorption of N_2_ on reduced ceria, we performed DFT calculations using surface and nanoparticle models. In some configurations, the nitrogen molecule was positioned perpendicular to the surface and remained bound via one of its nitrogen atoms to Ce^3+^ cations. Other configurations show the nitrogen molecule reorienting to a near-parallel position relative to the surface, binding via both nitrogen atoms to adjacent cerium cations. 

Table 2 summarizes the calculated binding energies of dinitrogen molecule in the studied model ceria systems, as well as the vibrational frequencies for ^15^N_2_ adsorbed via one nitrogen atom at Ce^3+^ sites. The N–N stretching modes for the adsorption where both nitrogen atoms are coordinated were expected to be IR-inactive. Interestingly, the binding energies for the surface parallel configurations on CeO_2_(100) and CeO_2_(110) planes were 0.10 to 0.20 eV higher in absolute value compared with those of the linear complexes. This increase can be attributed to the bonding mode and additional dispersion interactions present in these structures.

**Figure 8:**
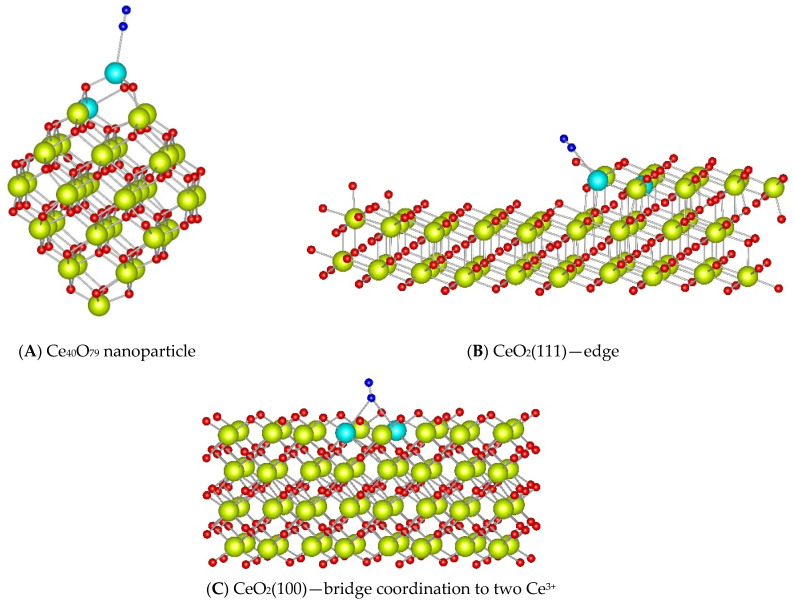
Complexes of N_2_ ligand adsorbed to Ce^3+^ cations located at the surface reduced by creating an O vacancy in Ce_40_O_80_ nanoparticle (**A**), CeO_2_(111) stepped (**B**), and CeO_2_(100) (**C**) surface models. Color coding: Ce^4+^—yellow, Ce^3+^—light blue, O—red, N—blue.

The calculated ^15^N_2_ vibrational frequencies for the complexes bound by one N-atom were as follows: (i) 2249 cm^−1^ for linear coordination to a Ce^3+^ cation in the nanoparticle model; (ii) 2247 cm^−1^ for adsorption at the edge of a stepped CeO_2_(111) surface; and (iii) 2241 cm^−1^ for bridge coordination to two Ce^3+^ cations in the CeO_2_(100) surface model (Figure 8).

These calculated frequencies help interpret the experimental FTIR data. The observed band at 2256–2255 cm^−1^ across all samples likely corresponds to ^15^N_2_ adsorbed at exposed Ce^3+^ sites, such as at corners or edges, with calculated frequencies of 2249–2247 cm^−1^. The second experimental band at 2254–2253 cm^−1^ is consistent with bridge-coordinated ^15^N_2_ on the CeO_2_(100) plane, which aligns with the calculated frequency of 2241 cm^−1^. Other surface sites, where the nitrogen molecule is bound through both nitrogen atoms, likely do not contribute to the observed IR spectra due to IR inactivity.

## 3. Discussion

This study presents a comprehensive analysis of ^15^N_2_ adsorption on reduced ceria, combining experimental techniques (primarily in situ FTIR spectroscopy and TPR) with DFT modeling. The results are compared with those obtained for stoichiometric (oxidized) ceria. Below, we discuss the spectral features of adsorbed dinitrogen, the potential active sites for its adsorption on reduced ceria and the feasibility of using N_2_ as a probe molecule for ceria-containing systems. As this is a pioneering study, some questions remain open.

### 3.1. Spectral Features of Adsorbed Dinitrogen

In their seminal work, Zecchina et al. [49] discussed the reference frequency for adsorbed dinitrogen. They selected a Raman value of 2324 cm^−1^, corresponding to ca. 2248 cm^−1^ for ^15^N_2_. When the N_2_ molecule is “on-top” adsorbed on a cationic site that is an electrostatic acid, it is polarized and the N–N stretching mode is activated in the IR spectra. This also leads to an increase in the N–N stretching frequency due to the Stark effect. The extinction coefficient also increases with polarization.

The adsorption causes frequency shift and the band intensity correlates with the polarization force of the cation. For example, in alkali metal exchanged MOR zeolites, the ν(NN) shift increases in the sequence Cs^+^ < Rb^+^ < K^+^ < Na^+^ < Li^+^, ranging from 5 cm^−1^ (Cs^+^) to 16 cm^−1^ (Li^+^) [49]. A moderate increase in extinction coefficient with the polarizing force of the cation is also observed [49,74]. Notably, the interaction of N_2_ with Ce^3+^ and Ce^4+^ cations as well as with OH groups is also essentially electrostatic.

^15^N_2_ also interacts with OH groups. Since these groups usually have a lower polarizing ability than cations, the observed stretching frequency of adsorbed dinitrogen is also lower. Another possibility is for ^15^N_2_ to interact with basic sites [75]. In this case, the stretching frequency of the adsorbed molecule is also low. The interaction of ^15^N_2_ with hydroxyls gives rise to a weak band around 2248 cm^−1^, with a possible contribution of ^15^N_2_ adsorbed on basic sites.

However, the adsorption configuration may differ. Small diatomic molecules can bridge two cations in two ways: via one atom or via both atoms. The former configuration has already been reported for CO and N_2_ on oxidized ceria [24,59,76], as well as for CO on reduced ceria [25]. The results of the present study suggest that this adsorption mode is also likely for N_2_ on reduced ceria. In this case, the N_2_ stretching frequency depends on the electrostatic field but does not directly reflect the Lewis acidity, as the probe interacts with two cations simultaneously.

Another potential configuration involves the N_2_ molecule lying parallel to the surface and bridging two cations equally. In this idealized arrangement, the molecule is not polarized in the direction parallel to the surface and is thus IR-inactive. Small symmetry violations can lead to the appearance of IR bands, which will however be of negligible intensity. The same applies to cases in which N_2_ bridges two cations situated on two different planes.

### 3.2. Reduction of Ceria

The most important feature of ceria is its ability to reversibly lose oxygen under reducing conditions, simultaneously converting Ce^4+^ ions to Ce^3+^ without disrupting the robust crystal structure. This process yields materials with the general formula CeO_2−x_ (0 < x < 0.28) [1]. Reduction can also occur in vacuum, but only at high temperatures or upon irradiation. Conversely, in an oxidizing environment, e.g., at ambient conditions, these cations are readily reoxidized back to Ce^4+^.

Our results on the characterization of the non-reduced samples support this understanding. Neither IR spectroscopy (Figure 2) nor Raman spectroscopy (Figure S1) detected Ce^3+^ sites. A small amount of Ce^3+^ cations was detected by XPS and, as noted earlier, they likely originated from a slight reduction during the measurement process [64]. In contrast, the presence of Ce^3+^ in all reduced samples was unambiguously confirmed by an IR band observed at 2133–2095 cm^−1^, which corresponds to the ^2^F_5/2_ → ^2^F_7/2_, the spin-orbital electronic transition of Ce^3+^. Another indication of the reduction was the bluish color of the reduced samples.

It is well established that the reduction of pure ceria with H_2_ occurs in two stages. Initially, surface (and probably some subsurface) Ce^4+^ cations are reduced at temperatures below 900 K [14,21,23,27,38,44,45,46,61,62,63]. At higher temperatures, reduction of the bulk begins. While the temperature required for bulk reduction is largely independent of sample morphology, surface reduction is influenced by the crystallite habitus, as different surface structures exhibit varying reducibility. In any case, the two-stage reduction allows obtaining samples in which only the surface sites are reduced.

For particles larger than 5 nm, surface-only reduction has a weak impact on the overall stoichiometry, which remains close to CeO_2_. For instance, in the CeO_2_-NP sample with a specific surface area of 29 m^2^ g^−1^, assuming an idealized morphology enclosed by {111} facets (with a surface cerium cation density of ~3.83 atoms nm^−2^), about 3.2% of the cerium cations were located at the surface. Upon their full reduction, the stoichiometry changed to CeO_1.99_. The lower value we obtained suggests that some subsurface reduction also took place, most probably for the (111) plane. The effect of surface reduction on the stoichiometry was more pronounced in the case of the CeO_2_-NR sample, which had a significantly higher specific surface area (110 m^2^ g^−1^).

The data in Table S1, derived from TPR measurements, indicate that the stoichiometry of the reduced sample is consistent with expectations based on surface reduction, and even suggest some degree of subsurface reduction. Given that the reduction for the IR experiments was conducted under more aggressive conditions (100 mbar H_2_ for 30 min), we can reasonably conclude that all surface Ce^4+^ cations were converted to Ce^3+^. Indeed, adsorption of ^15^N_2_ on the reduced sample revealed no bands attributable to Ce^4+^ sites on the {100} and {110} facets or on edge sites. However, we cannot completely rule out the presence of a small number of residual Ce^4+^ cations on the {111} facets.

### 3.3. N_2_ Adsorption Sites on Reduced Ceria

A general observation in this study is that the intensity of the bands of ^15^N_2_ adsorbed on reduced samples was consistently lower than that on their oxidized counterparts. Furthermore, the observed bands were positioned between those found with oxidized ceria. Since the molar extinction coefficient for the ^15^N_2_ stretching modes decreases only moderately with decreasing wavenumber, these findings suggest a decrease in the number of N_2_ adsorption sites after sample reduction. As the total number of surface-exposed cations does not decrease upon reduction, this implies that some Ce^3+^ cations are too weakly acidic to form adducts with dinitrogen.

Several types of adsorption sites can be considered on reduced ceria: (i) OH groups (ii) basic sites; (iii) surface Ce^3+^ cations on low-index planes such as (100), (110), and (111); (iv) Ce^3+^ cations at defects, including edges and corners (especially relevant in nanomaterials); and (iv) residual to reduction Ce^4+^ sites.

Three of these types are excluded from further discussion. First are the OH groups, which only interact with ^15^N_2_ at relatively high pressures and the adducts can be distinguished by the low wavenumber band at 2248 cm^−1^. Second are the basic sites, evidenced on some oxides by CO adsorption [77]. If they adsorb N_2_, the complexes formed should be monitored by a component of the weak band around 2248 cm^−1^. Third, non-reduced Ce^4+^ sites, where experimental evidence indicates that all Ce^4+^ cations on the {100} and {110} facets are reduced. Moreover, CO adsorption studies suggest that the remaining Ce^4+^ cations on {111} facets, if any, are of a negligible amount. Thus, the focus shifts to Ce^3+^ sites on regular planes and at defect sites.

First we underline that, to explicitly attribute some IR bands to definite surface planes, experiments with monocrystals are required. However, the use of shape-controlled nanoparticles combined with DFT calculations allows making some reasonable conclusions.

Reduction affects the Lewis acidity of cations through different mechanisms. Lowering the oxidation state of an isolated cation typically leads to reduced polarizing force due to the decrease in the positive charge and increase in the cationic radius. However, in oxide systems, cations are coordinated by oxygen anions, and reduction results in a lower coordination number. This should enhance the Lewis acidity. The resulting acidity depends on the interplay between these opposing effects. Based on our analysis of CO adsorption studies [25], we previously concluded that ceria reduction leads to (i) a sharp decrease in acidity for cations on {110} facets; (ii) a slight decrease on {100} facets; and (iii) a slight increase on {111} facets.

Cations at edges and defects are highly undercoordinated and remain the most active adsorption sites, despite some loss in acidity after reduction. Based on these considerations and on the DFT results, we assign the main ^15^N_2_ band at 2255 cm^−1^ to dinitrogen adsorbed at defect sites, primarily on crystal edges.

It was reported that the reduction of cations on the CeO_2_(111) plane leads to a slight increase in their Lewis acidity [25]. Thus, although the oxidized plane is inert towards adsorption, it is possible that the reduced facets adsorb N_2_. However, we do not support such assumption due to two reasons: (i) DFT calculations indicate low stability of Ce^3+^-N_2_ adducts on reduced CeO_2_(111) terrace and (ii) the 2255 cm^−1^ band is particularly weak in the CeO_2_-NP sample, which predominantly exposes the {111} facets.

The shoulder band at 2254 cm^−1^ is most pronounced for nanocubes, indicating it can be associated with the (100) facets. This aligns with DFT predictions of N_2_ bridging two Ce^3+^ cations with one nitrogen atom. This also explains the higher stability and lower frequency of the band at 2254 cm^−1^ compared with the main band at 2256 cm^−1^. Although the polarizing force is weaker, N_2_ is bound to two cations simultaneously, resulting in stronger binding. The relatively low band intensity can be explained by both a lower extinction coefficient (consistent with the lower frequency) and the adsorption geometry; if one N_2_ molecule bridges two cations, the number of adsorbed molecules is effectively halved relative to the number of available sites.

In summary, the specific arrangement of Ce^3+^ cations at the surface, including the exposure of {100}, {110}, and {111} facets, influences both the strength and the geometry of dinitrogen adsorption.

### 3.4. Potential Use of N_2_ as an IR Probe Molecule for Testing Surface of Ceria and Related Materials

The present results indicate that N_2_ has both advantages and limitations as a probe for ceria surfaces. The main limitation is its inability to interact with very weak Lewis acid sites. Therefore, a complete assessment of the Lewis acidity should combine N_2_ with another probe molecule, such as CO or pyridine.

Nonetheless, N_2_ offers important advantages. Its chemical inertness ensures that it does not form charged species upon adsorption, preserving the original surface speciation. Additionally, due to the weak nature of its interaction with the surface and the fact that no geminal species are formed, band positions are nearly independent of surface coverage. Consequently, differences in band maxima greater than 1 cm^−1^ can be considered significant. Thus, N_2_ can provide clear evidence for the presence of acidic Ce^4+^ and/or Ce^3+^ cations on surfaces subjected to different pre-treatments.

### 3.5. Future Directions

Since this is a pioneering study for the use of N_2_ as an IR probe for the surface of ceria and related materials, it leaves some open questions. Future works could focus on extending the reported findings here to more complex catalytic systems, including modified ceria (e.g., sulfated samples), doped ceria (e.g., with transition metals), mixed oxides (e.g., ceria-zirconia), and ceria with adsorbed species (e.g., peroxides and/or O_2_^−^). Additionally, investigations with single crystals could be essential to validate the conclusions drawn here.

## 4. Materials and Methods

### 4.1. Synthesis of the Samples

Two nanoscale ceria samples with distinct morphologies—cubes and rods—were synthesized using a hydrothermal method [9,18]. An aqueous solution of 5 g of Ce(NO_3_)_3_·6H_2_O (Fluka, Buchs, Switzerland, 99% purity) in 85 mL of water was added dropwise to 150 mL of a 36 wt.% NaOH solution (Merck, Darmstadt, Germany, 99% purity) under vigorous stirring. After forming a suspension, stirring continued for an additional 30 min before transferring the mixture to an autoclave.

To obtain nanocubes (designated CeO_2_-NC) and nanorods (CeO_2_-NR), the autoclave was maintained at 453 K and 373 K, respectively, for 24 h. Following the hydrothermal process, the suspensions were centrifuged, the precipitates were thoroughly rinsed with deionized water, dried at 393 K, and then calcined at 673 K for 2 h.

Nanopolyhedra (CeO_2_-NP) were prepared by calcining a portion of the CeO_2_-NC sample at 923 K for 1 h. It is well-documented that annealing ceria nanocubes at temperatures exceeding 873 K induces surface reconstruction resulting in the formation of {111} facets [24,27,60].

### 4.2. Characterization Techniques

FTIR spectra were recorded in situ using a Nicolet 6700 FTIR spectrometer equipped with a liquid nitrogen-cooled MCT-A detector (Thermo Scientific, Waltham, MA, USA), with 64 scans collected at a spectral resolution of 2 cm^−1^. Self-supporting pellets (~10 mg cm^−2^) were prepared by pressing powdered samples and analyzed in a custom-built glass IR cell allowing measurements from ambient temperature down to 100 K. The IR cell was connected to a vacuum-adsorption system, maintaining a residual pressure below 10^−3^ Pa. The samples were initially activated by heating in 100 mbar of O_2_ at 773 K for 30 min, followed by evacuation at the same temperature for an additional 30 min. These samples will be referred to further on as oxidized samples. To obtain reduced materials, the oxidized samples were subjected to reduction in 100 mbar of H_2_ for 30 min, followed by evacuation for 30 min, all this at 773 K. Finally, adsorption of ^15^N_2_ was performed at 100 K and an initial equilibrium pressure of 0.5 mbar, followed by evacuation. To ensure efficient thermal conductivity, helium (2 mbar) was introduced into the IR cell prior to the addition of ^15^N_2_.

For the in situ FTIR experiments, the following gases and adsorbates were used: ^15^N_2_ (Sigma-Aldrich, Darmstadt, Germany, 98 at. %), O_2_ (Messer, Clifford, Germany, 99.999%), H_2_ (Messer, >99.999%), and He (Messer, 99.999%). Prior to use, both ^5^N_2_ and He were further purified by passing through a liquid nitrogen trap.

Temperature-programmed reduction experiments were performed using ChemBET TPR/TPD (Quantachrome Instruments Co., Ltd., Boynton Beach, FL, USA) apparatus equipped with a thermal conductivity detector and using a 5% H_2_/Ar mixture (Messer) at a flow rate of 20 mL min^−1^ as a reducer. Before the experiments, the samples were heated to 723 K in a flowing stream of 5% O_2_/He (Messer) and then cooled down to room temperature. TPR curves were recorded while reducing the samples and increasing the temperature from 300 to 1273 K at a ramp rate of 10 K min^−1^. For separate TPR runs, the samples were first reduced to 773 K for 1 h and then cooled in an Ar flow before the TPR run.

Raman spectra were obtained with a Renishaw inVia™ confocal Raman microscope (Renishaw, Wotton-under-Edge, UK) using. The wavelength of the laser was 532 nm.

The high-resolution transmission electron microscopy patterns were obtained with a JEOL 2100 transmission electron microscope (JEOL Ltd., Tokyo, Japan) at an accelerating voltage of 200 kV.

Powder X-ray diffraction (XRD) analysis was performed on a Bruker D8 Advance diffractometer (Bruker AXS, Berlin, Germany) exploring a CuKα radiation.

The textural characteristics of the samples were determined by low-temperature nitrogen adsorption using a static volumetric Quantachrome NOVA 1200e apparatus (Quantachrome Instruments Co., Ltd., Boynton Beach, FL, USA).

### 4.3. DFT Studies

The periodic plane wave density functional theory (DFT) calculations were carried out using VASP code [78,79,80]. We used PW91 exchange–correlation functional [81] in combination with the empirical correction for dispersion interaction (PW91+D2) [82]. For the proper localization of the Ce4f electrons in the modeled systems containing Ce^3+^ cations, we employed on-site Coulombic correction with U = 4 eV [83,84]. We set the kinetic energy cut-off to 415 eV. The description of the core valence–electron interactions was performed employing the projector-augmented wave scheme [85]. The geometry of the models was relaxed until the maximum forces on each atom became lower than 0.02 eV/Å. The Brillouin zone consists of the Γ point only.

The binding energy of N_2_ (BE) to Ce^4+^ or Ce^3+^ cations was calculated as follows:

BE = E(N_2_/CeO_2−x_) − E(CeO_2−x_) − E(N_2_) (1)

where E(N_2_/CeO_2−x_) is the electronic energy of the adsorption complex of N_2_ to a cerium cation from the corresponding reduced ceria system, E(CeO_2−x_) is the energy of the ceria system, and E(N_2_) is the energy of the N_2_ molecule in the gas phase. Thus, the negative values of BE correspond to an attractive interaction. The calculated frequencies for ^15^N_2_ were scaled by 0.9668, as described previously [59], to compensate for the difference between the experimental Raman frequency, 2331 cm^−1^ [86], and the calculated value of isolated ^14^N_2_, 2411 cm^−1^.

We employed four models of ceria to study the N_2_ adsorption on reduced ceria systems, which are based on the structures reported earlier [24]—slab models of CeO_2_(111) surface with a step and regular surfaces of CeO_2_(100) and CeO_2_(110), as well as a Ce_40_O_80_ nanoparticle model. From those models we produced reduced structures by the removal of one or two oxygen atoms, thus reducing two or four Ce^4+^ to Ce^3+^ cations.

## 5. Conclusions

The low-temperature (100 K) adsorption of ^15^N_2_ on reduced ceria leads to the formation of the following surface species:

- Species characterized by an IR band at 2256–2255 cm^−1^. We attribute this band to ^15^N_2_ coordinated linearly to a corner or edge Ce^3+^ site on the ceria surface.
- Species characterized by an IR band at 2254–2253 cm^−1^. We assign this band to ^15^N_2_ bridging two Ce^3+^ cations on the CeO_2_{100} facets by one nitrogen atom.
- ^15^N_2_ polarized by surface OH groups of ceria. This adsorption form is only observed in the presence of gas-phase ^15^N_2_ and appears at 2248 cm^−1^ in the IR spectra. The contribution of ^15^N_2_ adsorbed on basic sites to this band is not excluded.
- Due to their very weak acidity, the Ce^3+^ sites on the regular CeO_2_{110} and CeO_2_{111} facets are considered not to form detectable complexes with ^15^N_2_.

Therefore, IR spectroscopy of ^15^N_2_ adsorbed at low temperature can be used to identify and monitor acidic Ce^3+^ sites and to distinguish them from Ce^4+^ cations.

## Figures and Tables

**Table 1:** Basic characteristics of the ceria samples studied.

Sample	Crystallite Shape	Dominant Facets	Average Crystallite Size [nm] *	S_BET_ [m^2^ g^−1^]	Pore Volume [cm^3^ g^−1^]	Average Pore Diameter [nm]
CeO_2_-NC	cubes	{100}	27.2	31	0.17	22
CeO_2_-NP	polyhedra	{111}	29.2	29	0.19	26
CeO_2_-NR	rods	{110}	6.3	110	0.46	17

* Crystallite size was estimated from XRD using the Scherrer equation.

**Table 2:** Calculated binding energies (BE) of ^15^N_2_ molecule in the studied model ceria systems and vibrational frequencies for ^15^N_2_ adsorbed via one of the nitrogen atoms at Ce^3+^ centers.

Model	BE [eV]	Calculated Frequency [cm^−1^]	Experimental Frequency [cm^−1^]
Gas phase	−	2252	2252
Nanoparticle	−0.27	2249	2256–2255
CeO_2_(111)—edge	−0.34	2247	2256–2255
CeO_2_(100)—bridge	−0.40	2241	2254–2253
CeO_2_(100)—parallel	−0.60	2025, 2018	−
CeO_2_(110)—parallel	−0.50	2083	−

## Data Availability

Data will be made available upon request.

## Supplementary Materials

The following supporting information can be downloaded at: https://www.mdpi.com/article/10.3390/molecules30153100/s1. Figure S1. Raman spectra of the samples; Figure S2. FTIR spectra of the Ce^3+^ ^2^F_5/2_ → ^2^F_7/2_ electronic transition band; Figure S3. FTIR spectra of doses of ^15^N_2_ successively adsorbed on reduced ceria nanorods; Figure S4. IR spectral changes occluding after dosing CO to the ^15^N_2_-CeO_2_-NR system; Figure S5. IR spectral changes occurring after dosing O_2_ to the ^15^N_2_-CeO_2_-NR system. Table S1. Consumption of H_2_ during reduction of ceria samples.
