# Supplementary material for: FTIR Detection of Ce3+ Sites on Shape-Controlled Ceria Nanoparticles Using Adsorbed 15N2 as a Probe Molecule
Source: Molecules. 2025 Jul 24;30(15):3100. doi: 10.3390/molecules30153100 (PMC12348644; doi:10.3390/molecules30153100)
Supplement: Supplementary file 1 [file molecules-30-03100-s001.zip › molecules-3741314-supplementary.pdf]

Supporting Information

FTIR Detection of Ce^{3+} Sites on Shape-Controlled Ceria Nanoparticles Using Adsorbed $^{15}N_2$ as a Probe Molecule

Kristina K. Chakarova, Mihail Y. Mihaylov, Bayan S. Karapenchev, Nikola L. Drenchev, Elena Z. Ivanova, Georgi N. Vayssilov, Hristiyan A. Aleksandrov and Konstantin I. Hadjiivanov

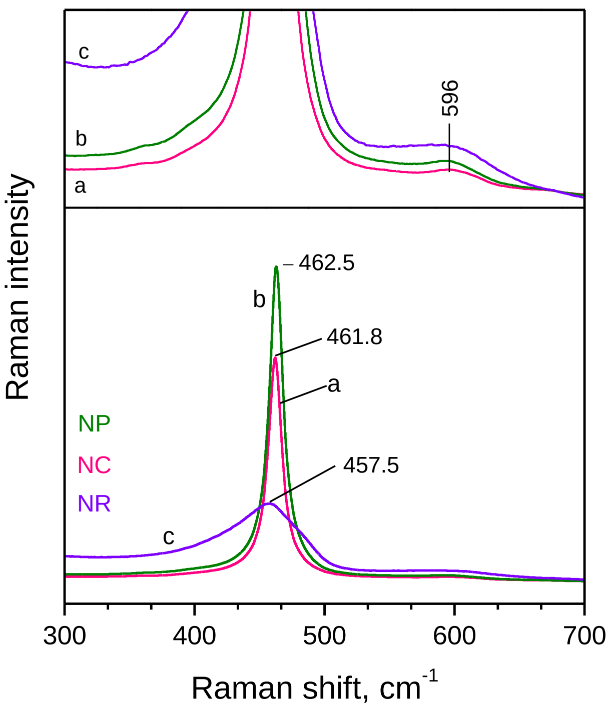

Figure S1. Raman spectra of CeO_2-NC (a), CeO_2-NP (b) and CeO_2-NR samples. Spectrum (c) is multiplied by 3.

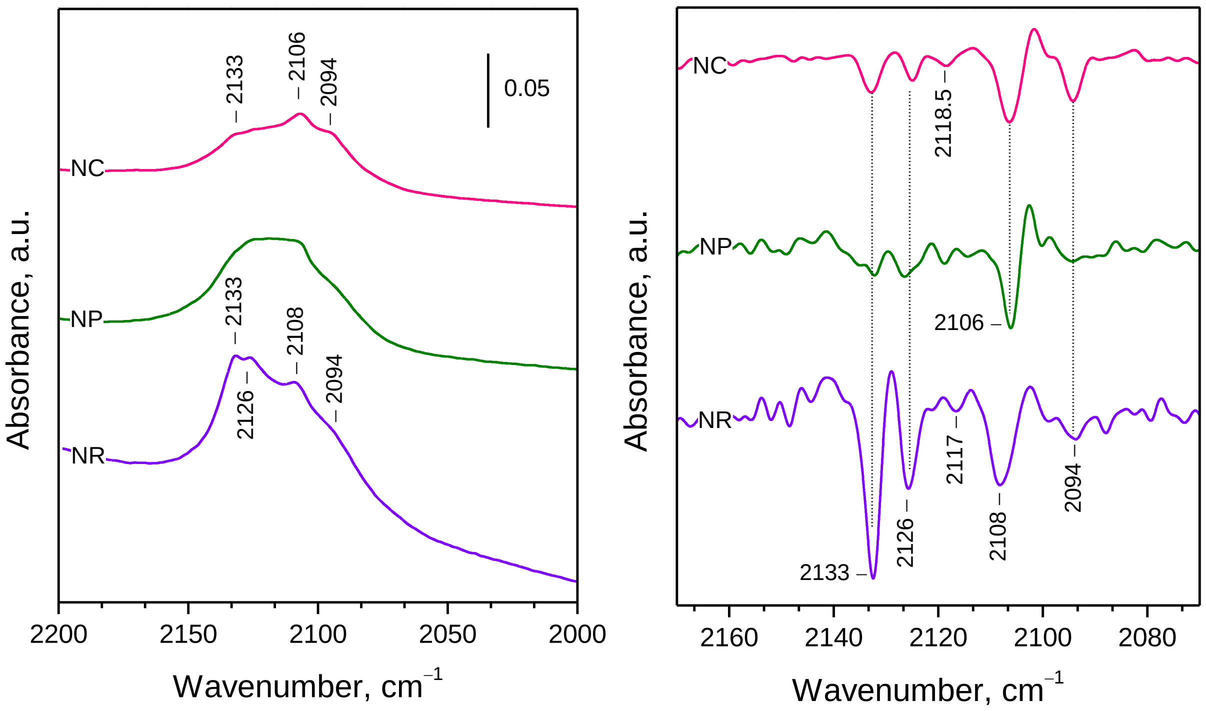

Figure S2. Ce^{3+} Ce^{3+} $^{2}F_{5/2} \rightarrow {}^{2}F_{7/2}$ electronic transition bands and their derivatives for the different samples studied. Spectra are registered at 100 K.

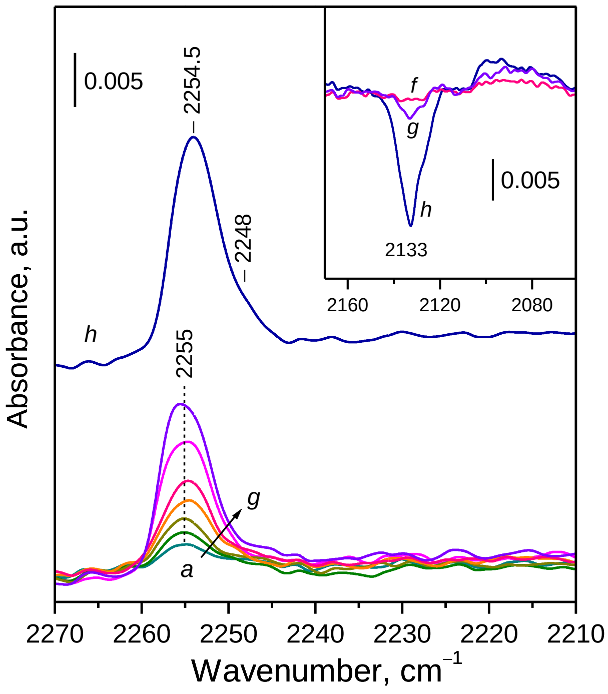

Figure S3. FTIR spectra of small doses of $^{15}N_2$ successively adsorbed on reduced CeO_2-NR sample (1-g) and in the presence of 0.5 mbar $^{15}N_2$ (h).

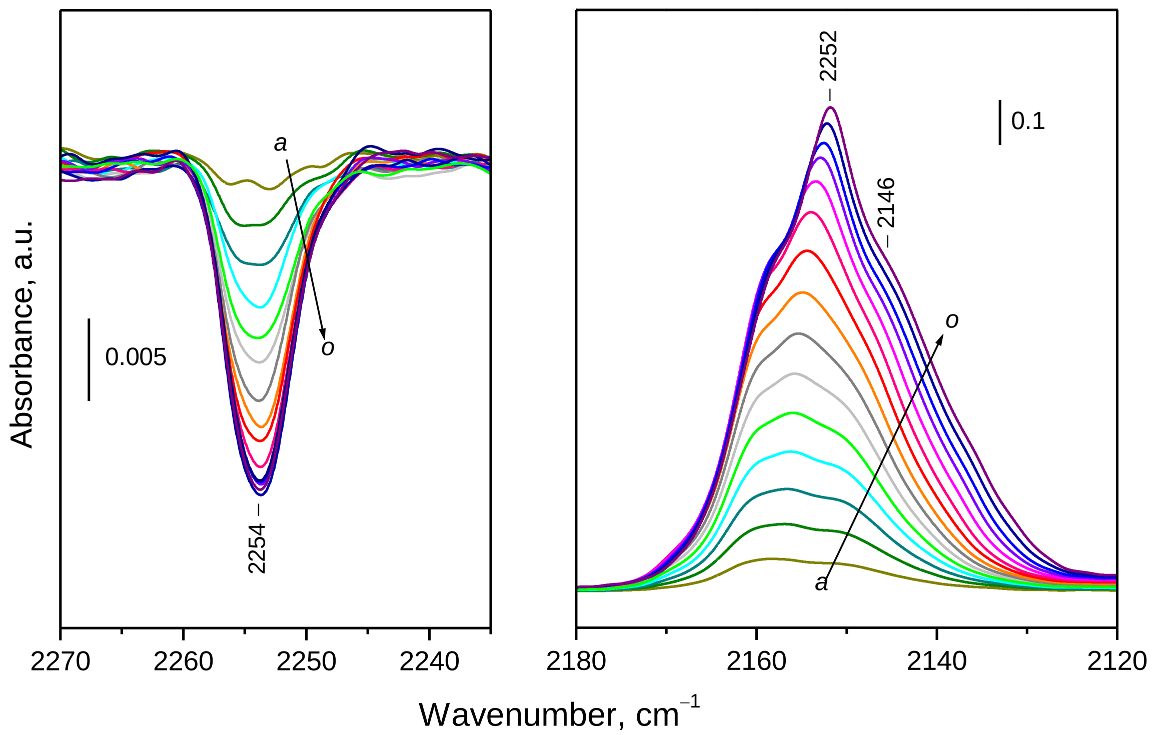

Figure S4. Changes in the IR spectra of the $^{15}N_2$ – CeO_2-NR system after successive addition of small doses on CO at 100 K (a-o).

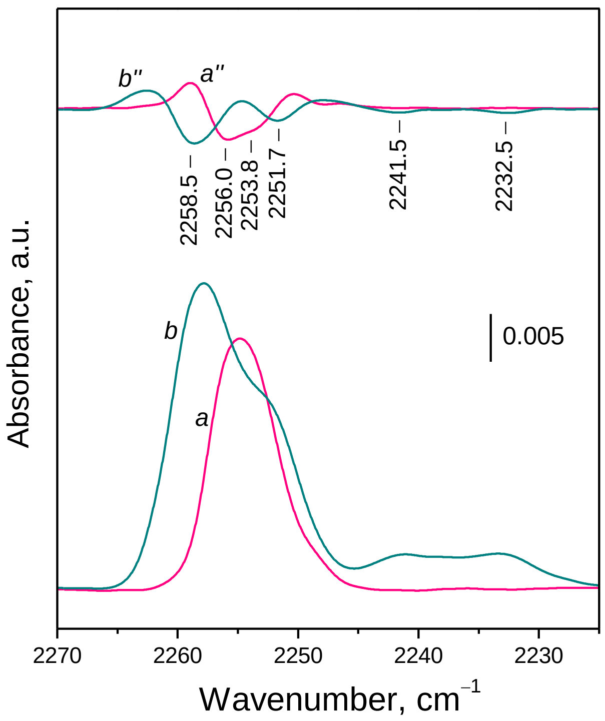

Figure S5. IR spectra recorded after adsorption of $^{15}N_2$ (0.5 mbar, 100 K) on CeO_2-NC (a) and after addition of a portion of oxygen to the system (b). The spectra are background corrected.

Table S1. Consumption of H_2 during the TPR experiments with the calcined samples and the formal stoichiometry achieved.

Sample	H_2 consumption between 373 and 773 K, μmol H_2 g^{-1}	At. % of Ce^{4+} cations reduced	Stoichiometry	H_2 consumption for the peaks below 900 K, μmol H_2 g^{-1}	At. % of Ce^{4+} cations reduced	Stoichiometry
CeO_2-NC	125.4	4.31	$CeO_{1.978}$	152.0	5.23	$CeO_{1.974}$
CeO_2-NP	139.4	4.80	$CeO_{1.976}$	344.7	11.86	$CeO_{1.941}$
CeO_2-NR	249.9	8.60	$CeO_{1.957}$	288.2	9.91	$CeO_{1.950}$
